# Characterization and Comparative Analysis of Complete Mitogenomes of Three *Cacatua* Parrots (Psittaciformes: Cacatuidae)

**DOI:** 10.3390/genes12020209

**Published:** 2021-01-31

**Authors:** Jung-Il Kim, Thinh Dinh Do, Yisoo Choi, Yonggu Yeo, Chang-Bae Kim

**Affiliations:** 1 Department of Biotechnology, Sangmyung University, Seoul 03016, Korea; kim2429@gmail.com (J.-I.K.); deepblue.th@gmail.com (T.D.D.); choi.yisoo.hi@gmail.com (Y.C.); 2 Conservation and Health Center, Seoul Zoo, Gwacheon 13829, Korea; withyonggu@seoul.go.kr

**Keywords:** *Cacatua alba*, *Cacatua galerita*, *Cacatua goffiniana*, Cacatuidae, complete mitogenome, phylogeny

## Abstract

*Cacatua alba*, *Cacatua galerita,* and *Cacatua goffiniana* are parrots of the family Cacatuidae. Wild populations of these species are declining with *C. alba* listed by the International Union for the Conservation of Nature and Natural Resources (IUCN) as Endangered. In this study, complete mitogenomes were sequenced for a comparative analysis among the *Cacatua* species, and a detailed analysis of the control region. Mitogenome lengths of *C. alba,* *C. galerita,* and *C. goffiniana* were 18,894, 18,900, and 19,084 bp, respectively. They included 13 protein coding genes, two ribosomal RNA genes, 24 transfer RNA genes, three degenerated genes, and two control regions. Ten conserved motifs were found in three domains within each of the two control regions. For an evolution of duplicated control regions of *Cacatua*, domain I and the 3′ end of domain III experienced an independent evolution, while domain II and most of the regions of domain III was subjected to a concerted evolution. Based on a phylogenetic analysis of 37 mitochondrial genes, the genus *Cacatua* formed a well-supported, monophyletic, crown group within the Cacatuidae. Molecular dating results showed that *Cacatua* diverged from other genera of Cacatuinae in the middle of Miocene.

## 1. Introduction

The cockatoos (family: Cacatuidae) are characterized by their comparatively large size, a robust reinforced skull, predominantly white or pink, or in some species black or grey, plumage, and erectile, and often colorful, crests. [1]. Twenty-one species of Cacatuidae have been identified and classified into five genera: *Cacatua*, *Calyptorhnchus*, *Eolophus*, *Nymphicus,* and *Probosciger*. Most of them live in Australia while others inhabit New Guinea or the islands of Wallacea, Melanesia, and the Philippines [2]. Their habitats vary from mangrove forests to subalpine woodlands [2]. Among all genera, *Cacatua* is the largest genus of family Cacatuidae, including 12 species. All *Cacatua* species have predominantly white plumage with the plumage of *Cacatua moluccensis* and *Cacatua leadbeateri* infused with pink. [2].

Cockatoos are popular pets and are listed among the top ten traded species in Medan and Sumatran markets [3]. Much of this trade in cockatoos throughout Southeast Asia is illicit. Cockatoos are protected from extinction and illegal trades by many conventions and conservation bodies such as International Union for the Conservation of Nature and Natural Resources (IUCN) and Convention on International Trade in Endangered Species of Wild Fauna and Flora (CITES). According to the IUCN Red List of Threatened Species, the wild populations of ten *Cacatua* species, including the three species described in this study, are declining, especially *Cacatua haematuropygia* and *Cacatua sulphurea,* both of which are listed as Critically Endangered. The predominantly Sulawesi species *C. alba* is considered Endangered, while three other species (*C. goffiniana*, *Cacatua opthalmica*, and *Cacatua moluccensis*) are considered Threatened or Near Threatened (https://www.iucnredlist.org) [4]. According to the CITES checklist, four *Cacatua* species (*C. goffiniana*, *C. haematuropygia*, *C. moluccensis,* and *C. sulphurea*) are listed under Appendix I meaning that trade is prohibited. Another seven species are listed under Appendix II meaning that trade is closely controlled by means of export permits.

Mitochondrial genomes (mitogenomes) are an important source of genetic markers in phylogenetics and other evolutionary studies due to their general maternal inheritance and subsequent lack of recombination and mutation rate [5]. In addition, mitogenomes are also valuable markers for DNA barcoding and analysis of the population structure for conservation [6,7]. The most common markers for DNA barcoding were the mitochondrial cytochrome c oxidase subunit I gene (*cox1*) and cytochrome b gene (*cytb*) [8,9]. Moreover, the mitochondrial 12S and 16S ribosomal RNA genes were used for species identification in avian [10,11]. The *cox1* and control region (CR) of mitogenome were widely used in the study of the population structure in avian [12,13,14,15]. Vertebrate mitogenomes are considered highly conserved due to their similar gene content and order [16]. The recent reports indicate that the CR and nearby genes experience tandem duplication and degenerations of the duplicated copies. Duplicated CRs and adjacent genes in the parrot mitogenome were first found in the genus *Amazona* [17]. In addition, several gene arrangements of parrot mitogenomes including the duplicated region have been reported [18,19,20]. The CR is a crucial noncoding region that contains the origin of replication and transcription for the mitogenome [21,22] and is comprised of three domains carrying conserved motifs. Domain I includes extended termination associated sequences (ETAS) [23]. Domain II contains several conserved sequences as a box [24]. Domain III consists of conserved sequence blocks (CSB) 1 to 3 [25] and a bidirectional transcription promoter [19]. However, the structure and evolution of CRs of *Cacatua* species remain little known.

The extant Cacatuidae are the descendants of a comparatively ancient split within the parrots [20,26]. Despite the importance of the Cacatuidae in understanding the evolutionary history of the parrots, only mitogenomes of two species within the *Cacatua* species, the largest cockatoo genus, have been sequenced [20,26]. In this study, complete mitogenomes of *C. alba*, *C. galerita,* and *C. goffiniana* were sequenced, analyzed, and compared with the other available mitogenomes of *Cacatua* genus. Especially, the structure and evolutionary scenario of CRs of *Cacatua* species were analyzed. In addition, phylogenetic relationships and molecular dating within the family Cacatuidae were analyzed using data from 37 mitogenomes.

## 2. Materials and Methods

### 2.1. Ethics and Sampling

Ethical clearance for this study was approved by Seoul Zoo IACUC (number: 2019-001). All the sampling protocols were in accordance with the standards of this committee. Blood samples of *C. alba*, *C. galerita,* and *C. goffiniana* were obtained from Seoul Zoo and stored at −20 °C.

### 2.2. DNA Extraction and Molecular Identification

Total genomic DNA was extracted with a DNeasy Blood and Tissue Kit (Qiagen Inc., Hilden, Germany) following the manufacturer’s protocol. After DNA extraction, a partial mitochondrial cytochrome b (*cytb*) gene was amplified [27,28] using the following PCR protocol: Initial denaturation at 95 °C for 2 min followed by 35 cycles of denaturation at 95 °C for 45 s, annealing at 52 °C for 45 s, extension at 72 °C for 1 min, and a final elongation step at 72 °C for 5 min. Final PCR products were sequenced with an Agilent 2100 bioanalyzer (Agilent, Santa Clara, USA).

### 2.3. Library Preparation and Next Generation Sequencing

Total genomic DNA of 1 µg was obtained from each of the three species and fragmented using an S220 ultra sonicator (Covaris, Woburn, USA). The library preparation was performed using a MGIEasy DNA library prep kit (MGI, Shenzhen, China) according to the manufacturer’s instructions. The library was quantified with a QauntiFluor ssDNA System (Promega, Madison, USA). Sequencing was performed for a MGI paired-end library, and 150 bp paired-end reads were generated using a MGISEQ-2000 platform (MGI, Shenzhen, China).

### 2.4. Mitochondrial Genome Analysis

Mitogenome sequences of each species were assembled baiting with the complete mitogenomes of *C. moluccensis* (MH133972) [20] by MITObim [29]. Protein coding genes and ribosomal RNA genes were identified with the MITOS web server [30]. Transfer RNA genes were identified using the ARWEN web server [31]. Mitochondrial genomes were manually curated using the Geneious Prime 2019.2.3 software [32] and compared with the reported *Cacatua* genus mitogenomes. The region including the duplicated CR of approximately 3.5 kb was confirmed with primer sets (Supplementary Table S1) using the following PCR protocol: Initial denaturation at 95 °C for 2 min followed by 35 cycles of denaturing at 95 °C for 45 s, annealing at 52 °C for 45 s, extension at 72 °C for 1 to 2 min, and a final elongation step at 72 °C for 5 min. Skewness was calculated based on the composition of nucleotide sequences using the following formula: AT skew = [A − T]/[A + T], GC skew = [G − C]/[G + C] [33].

### 2.5. Control Region Analysis

Conserved motifs of CRs of the *Cacatua* genus were identified by comparing the three mitogenomes from this study to those from the same genus retrieved from GenBank (Table 1) [20]. CRs were aligned with MAFFT [34]. Conserved motifs were identified by comparison with the mitogenomes of *Amazona* genus [17,35]. A phylogenetic analysis using CRs was conducted with Cacatuidae mitogenomes having duplicated CRs (Table 1) [20]. The alignment for CRs was conducted with MAFFT [34]. In addition, the analysis of sequence similarity and phylogeny using each three domains of CRs of the *Cacatua* species were conducted. Poorly aligned regions were trimmed with GBlocks [36]. The GTR GAMMA I model for CRs, domain I and domain II dataset, and HKC GAMMA I model for domain III dataset were searched as a best-fit model with Akaike and Bayesian criteria in jModelTest [37]. A phylogenetic analysis was conducted with Maximum likelihood (ML) using RAxML v.8.2.11 [38] in the Geneious Prime 2019.2.3 software [32] and Bayesian inference (BI) using MrBayes v.3.2.7 [39]. In RAxML, the GTR GAMMA I model was used, and node supports were calculated with 1000 bootstrap replicates. In MrBayes, two independent sets of Markov chain Monte Carlo algorithms were run for 10,000,000 generations and sampled every 100 generations. The standard deviation of split frequencies reached below 0.01 after 45,600 generations for the CRs dataset, 54,000 generations for the domain I dataset, 28,000 generations for the domain II dataset, and 15,500 generations for the domain III dataset. After the 10,000,000 generations run, the mean standard deviation of split frequencies was 0.001898, 0.000348, 0.000473, and 0.000330 for CRs, domain I, domain II, and domain III dataset, respectively. The potential scale reduction factor (PSRF) was 1 for all four datasets. Convergence of the MCMC chain and appropriate burn-in were assessed with Tracer 1.7.1 [40]. Consensus trees were constructed after discarding 25% of initial trees as burn-in. The resulting trees were visualized and edited using FigTree v1.4.3 available at http://tree.bio.ed.ac.uk/software/figtree/ [41].

### 2.6. Phylogenetic Analysis and Molecular Dating

Eight mitogenomes of Cacatuidae and two mitogenomes of Strigopidae retrieved from GenBank, as well as three mitogenomes from the present study were used to determine phylogenetic relationships within the Cacatuidae (Table 1) [20,26,43]. Two mitogenomes were available on GenBank for each of three cockatoo species (*C. moluccensis*, *C. pastinator*, and *Cal. baudinii*). A previous study showed that each mitogenome of these species had one control region [26]. However, a recent study based on different PCR strategies suggested that these species have duplicated control regions [20], and we herein referred to these results in our investigation. A total of 37 genes (including 13 protein coding genes, two ribosomal RNA genes, and 22 transfer RNA genes) were used for the phylogenetic analysis of Cacatuidae. The Geneious Prime 2019.2.3 software was used to concatenate 37 genes of mitogenome [32]. The alignment was conducted with MAFFT [34] and suitable regions for the phylogenetic analysis were chosen with GBlocks [36]. The best partition schemes and best-fit model were searched in PartitionFinder 2 (Supplementary Table S2) [44]. In this procedure, each of the PCGs and rRNA genes was treated as a partition and all 22 tRNA genes were treated as a partition. The phylogenetic analysis was conducted by Maximum likelihood (ML) using RAxML v.8.2.11 [38] in the Geneious Prime 2019.2.3 software [32] and Bayesian inference (BI) using MrBayes v.3.2.7 [39]. In RAxML, the GTR GAMMA I model was used, and node supports were calculated with 1000 bootstrap replicates. In MrBayes, two independent sets of Metropolis-coupled Markov chain Monte Carlo algorithms were run for 10,000,000 generations and sampled every 100 generations. The standard deviation of split frequencies reached below 0.01 after 4000 generations. After the 10,000,000 generations run, the mean standard deviation of split frequencies was 0.000002. The potential scale reduction factor (PSRF) ranged from 1 to 1.002. Tracer 1.7.1 [40] was used to assess the convergence of the MCMC chain as well as the appropriate burn-in based on the ESS values (>200) and the trace plots. Bayesian posterior probability values and consensus trees were calculated after discarding 25% of initial trees as burn-in. The resulting trees were visualized and edited using FigTree v1.4.3 available at http://tree.bio.ed.ac.uk/software/figtree/ [41].

Divergence times with the Cacatuiae were estimated with BEAST v2.6.3 [45] based on 37 mitochondrial genes. A GTR GAMMA I model was used following recommendations of jModelTest [38] with the lowest values in Akaike (139,174.2) and Bayesian (139,459.5) criteria. The analysis was performed with a calibrated Yule model and a clock model as a relaxed clock log normal. A total of 15 mitogenomes of Cacatuidae, Psittacidae, and Strigopidae were retrieved from GenBank for analysis (Table 1) [19,20,26,42,43]. Two mitogenomes of Strigopidae were used as an outgroup in the phylogenetic reconstructions. The most recent common ancestor (MRCA) Cacatuidae and Psittacidae was set as normal distribution priors with a mean of 40.69 mya and a standard deviation of 6.5 mya and the MRCA of Cacatuidae was set as normal distribution priors with a mean of 27.85 mya and a standard deviation of 5.8 mya [26]. MCMC chains were run for 20 million generations and trees were sampled every 1000 generations. These trees were annotated with 25% burn-in with TreeAnnotator v.2.6.3 [45] and visualized with FigTree v1.4.3.

## 3. Results and Discussion

### 3.1. Mitogenome Structure and Composition

**Figure 1:**
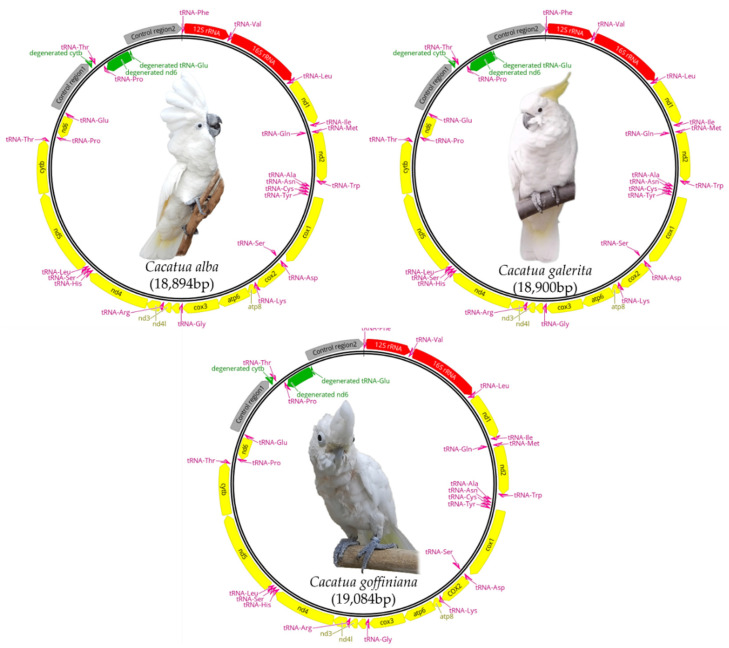
Maps of complete mitogenomes of three *Cacatua* species. Protein coding genes, rRNA genes, tRNA genes, degenerated genes, and control regions are marked with yellow, red, pink, green, and grey, respectively.

Mitogenomes of *C. alba*, *C. galerita,* and *C. goffiniana* were 18,894, 18,900, and 19,084 bp in length, respectively. *C. moluccensis* exhibited the shortest mitogenome (18,863 bp), while *C. goffiniana* from this study exhibited the longest one (19,084 bp). Mitogenomes of these three species included 13 protein-coding genes (PCGs), two ribosomal RNA genes, 24 transfer RNA genes, two control regions (CRs), and three pseudogenes caused by duplication (Figure 1 and Supplementary Tables S3–S5). Within the *Cacatua*, *C. moluccensis* had the same gene composition as these three species. However, *C. pastinator* had 14 PCGs and 25 tRNA [20]. The CR and nearby genes of mitogenome had a functional or degenerated second copy caused by duplication [17,19,20]. The duplicated functional copy of tRNA-Thr, tRNA-Pro and CR, and the degenerated *nd6* and tRNA-Glu of the three species described in this study, as well as in *C. moluccensis* [20]. In the case of *C. pastinator*, the functional copy of tRNA-Thr, tRNA-Pro, *nd6*, tRNA-Glu, and CR was duplicated [20]. Nucleotide compositions of mitogenomes were: 29.5% A, 31.6% C, 15.0% G, and 24.0% T for *C. alba*; 29.4% A, 31.8% C, 15.1% G, and 23.7% T for *C. galerita*; and 29.7% A, 31.3% C, 14.8% G, and 24.2% T for *C. goffiniana* (Table 2). A weakly positive AT skew and strongly negative GC skew were found in both whole sequences and PCGs of our three focal species and other Cacatuidae species (Supplementary Table S6). These were also found in most mitogenomes of Psittaciformes [19,20].

### 3.2. Protein Coding Genes

Thirteen PCGs were found in each of the three species investigated in this study. All the PCGs were encoded on a heavy (H) strand except *nd6*, which was encoded on a light (L) strand. The length of PCGs was the same in all *Cacatua* except *atp8* and *nd6*. The *atp8* in *C. galerita* was 165 bp long, three nucleotides shorter than that in the other *Cacatua* species, due to a deletion of three nucleotides in the middle of the gene. Similarly, *nd6* in *C. moluccensis* was 507 bp long than the twelve nucleotides shorter than the other *Cacatua* species and there was a deletion of nucleotides in the middle of the gene [20]. An intergenic nucleotide (C) was found in the middle of *nd3* genes. This was reported for 46 birds, including species in the order Psittaciformes and the intergenic nucleotide was not involved in translation [46]. Start codons were identical in 13 PCGs of the three species. There were three different start codons: GTG in *cox1*, ATA in *nd3,* and ATG in other 11 PCGs. Stop codons were also the same in these three species except *atp8*. These stop codons were: AGG in *nd1* and *cox1*; TAA in *cox2*, *nd4l*, *nd5,* and *cytb*; and TAG in *nd6*, TA in *nd2*, *atp6,* and *nd3*; and T in *cox3* and *nd4*. In the case of *atp8*, two types of stop codons were found: TAG in *C. alba* and *C. moluccensis* and TAA in *C. galerita, C. goffiniana,* and *C. pastinator*. 

**Figure 2:**
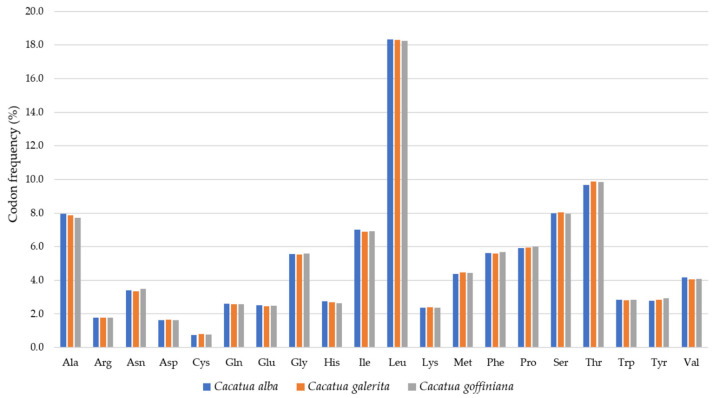
Codon frequencies of three focal species in this study.

For codon usage, Leucine (L) was the most frequent amino acid in these three species: 18.3% in *C. alba* and *C. galerita,* and 18.2% in *C. goffiniana.* Cysteine(C) was the least frequent amino acid accounting for 0.7% in *C. alba* and 0.8% in *C. galerita* and *C. goffiniana* (Figure 2). In the case of *C. moluccensis* and *C. pastinator*, the most frequent amino acid was also leucine (L): 18.3% in *C. moluccensis* and 18.0% in *C. pastinator* [20]. The least frequent amino acid was cysteine (C): 0.8% in *C. moluccensis* and *C. pastinator* [20].

### 3.3. Ribosomal and Transfer RNA Genes

Two ribosomal RNA genes in mitogenomes of these three species were encoded on the H-strand. The 12S rRNA genes were located between tRNA-Phe and tRNA-Val and the length ranged from 966 to 969 bp. The 16S rRNA genes were located between tRNA-Val and tRNA-Leu and the length ranged from 1571 to 1574 bp. The rRNA genes were the most GC-rich in mitogenomes of the three focal species (48.0%~49.0%).

A total of 24 tRNA genes were found in mitogenomes of the three focal species. The longest tRNA was tRNA-Leu in the upstream of *nd1*, measuring 76 bp for *C. galerita* and *C. goffiniana*. The shortest one was tRNA-Phe from *C. alba*, measuring 64 bp. Of the 24 tRNAs, there were two copies of tRNA-Leu, tRNA-Ser, tRNA-Pro, and tRNA-Thr found in three focal species. Two copies of tRNA-Leu and Ser with different anticodons were commonly found in animal mitogenome [16]. Two copies of tRNA-Leu and tRNA-Ser had different anticodons: tRNA-Leu contained TAA and TAG and tRNA-Ser had TGA and GCT. The sequence similarity among these two orthologous tRNA genes was higher than among the paralogous genes. In contrast, tRNA-Pro and tRNA-Thr possessed the same anticodon across all three focal species: TGG for tRNA-Pro and TGT for tRNA-Thr. In these two tRNA genes, the sequence similarity among the paralogous genes was higher than that among the orthologous genes. These results supported that two copies of tRNA-Pro and tRNA-Thr might be caused by duplication in the mitogenome.

### 3.4. Degenerated Genes

In this study, three degenerated genes were found in three species (Table 3). A degenerated *cytb* was found between the first control region and the second tRNA-Thr. Its length ranged from 113 to 115 bp in all three focal species. This degenerated gene was highly similar to the 3′ region of the functional *cytb* gene. The sequence similarity among paralogous genes was 92.1% for *C. alba*, 88.7% for *C. galerita,* and 97.3% for *C. goffiniana*. A 115 bp-long degenerated partial *cytb* was also found in *C. moluccensis* and *C. pastinator* and the sequence similarity among the paralogous genes of each species were 92.2% and 99.1%, respectively [20]. The degenerate *nd6* exhibited a high GA ratio region in all three focal species, with the length ranging from 650 to 680 bp. This gene had a high sequence similarity with a paralogous functional *nd6* ranging from 90.8% to 92.9%. The degenerate *nd6* exhibited an insertion in the 5′ region ranging from 126 to 159 bp with a GA ratio of 69.1% to 78.3%. In addition, the degenerate *nd6* showed a 95.0% sequence similarity with functional *nd6* and carried an insertion of 136 bp GA rich sequences in *C. moluccensis* [20]. Degenerate tRNA-Glu sequences were less similar to the functional tRNA-Glu ranging from 41.5% to 50.0%, and the lengths were 50 bp in *C. alba* and *C. galerita* and 51 bp in *C. goffiniana*. Degenerate tRNA-Glu was 33 bp in length and 60.6%, similar to the functional tRNA-Glu in *C. molussensis* [20]. This degenerate tRNA-Glu is likely nonfunctional since it has no cloverleaf secondary structure. *C. pastinator* possessed two copies of both a fully functional *nd6* and tRNA-Glu [20].

### 3.5. Control Regions

In the present study, mitogenomes of the three species included two CRs. These duplicated CRs could affect the energy production by facilitating the transcription and replication each time compared to nonduplication [47,48]. The length of CR1 was 1185 bp in *C. alba*, 1184 bp in *C. galerita,* and 1317 bp in *C. goffiniana*. The length of CR2 was 1242 bp in *C. alba*, 1246 bp in *C. galerita,* and 1272 bp in *C. goffiniana*. The lengths of CR1 and CR2 were 1186 and 1243 bp in *C. moluccensis* and 1322 and 1360 bp in *C. pastinator*, respectively [20]. In other parrots, the first CR was commonly shorter than the second CR. However, the opposite was found in several species of Psittacidae and Psittaculidae [17,19,45,49,50]. Paralogous CRs were more similar than orthologous CRs. The sequence similarity of paralogous CRs was 93.5% in *C. alba*, 94.3% in *C. galerita,* and 95.2% in *C. goffiniana*. The sequence similarity was 88.9% for orthologous CR1 and 87.3% for CR2 within the *Cacatua* genus.

**Figure 3:**
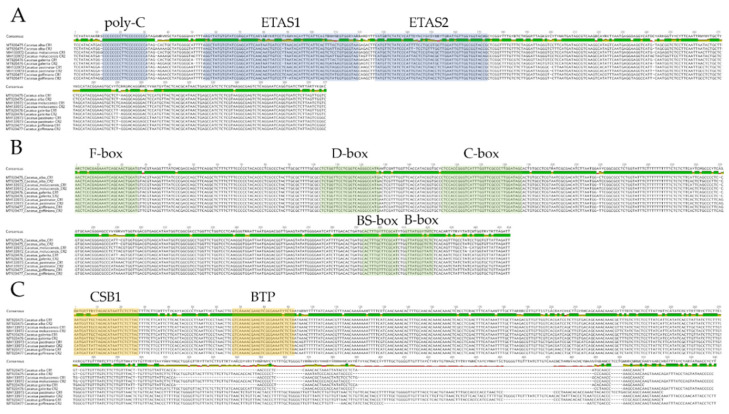
Ten conserved motifs in control regions of the *Cacatua* genus. (**A**): Domain I; (**B**): Domain II; (**C**): Domain III.

For the first time, conserved motifs in the *Cacatua* species were characterized and analyzed. A total of 10 conserved motifs were found in CRs of *Cacatua* based on a comparison with both paralogous and orthologous CRs (Figure 3 and Supplementary Table S7). Domain I contained poly-C with an identical TT in the middle of C repeats. Most of the poly-C chain including the first and second CRs of the *Cacatua* genus was CCCCCCCCTTCCCCCCCC. However, for the first CR of *C. alba*, an additional C was observed in the 5′ region (CCCCCCCCCTTCCCCCCCC). ETAS1 and ETAS2 were also present in domain I, showing 85.2% and 83.3% similarities, respectively. Domain II contained F, D, C, bird similarity (BS), and a B box with similarities of 96.6%, 98.6%, 100%, 96.4%, and 94.1%, respectively. Domain III contained a conserved sequence block (CSB) 1 with a 91.4% sequence similarity. CSB2 and CSB3 were not found in any of the three *Cacatua* species, although they did not occur in either of several avian species [24]. Moreover, a palindromic bidirectional transcription promoter (BTP) was present downstream of CSB1. In the domestic chicken, BTP is located downstream of CSB1 [51]. *Amazona* also has a similar region [35]. Although tandem repeats were found in domain III of *Amazona* genus [17,35], there were no tandem repeats in the *Cacatua* genus.

Most of the paralogous CRs were clustered together in the phylogenetic trees using CRs of Cacatuidae (Supplementary Figures S1 and S2). A high degree of sequence similarity and phylogenetic relationships between paralogous CRs were also found not only in Psittaciformes [17,20], but also in other avian orders [52,53,54,55,56,57,58,59,60,61]. These revealed that duplicated CRs in the avian mitogenomes are subjected to a concerted evolution [17,20,52,53,54,55,56,57,58,59,60,61]. Under a concerted evolution, CRs tend to homogenize, while other duplicated genes were degenerated in most of the *Cacatua* species examined in the study. However, the evolution of duplicated CRs in the *Cacatua* species is complicated. Among the five *Cacatua* species examined in this work, paralogous CRs of *C. alba*, *C. galertia*, *C. moluccensis,* and *C. pastinator* were clustered, while paralogous CRs of *C. goffiniana* were not clustered in phylogenetic trees. This phenomenon was also found in the *Amazona* species (order: Psittaciformes) [19] and two families, Aegithalidae and Zosteropidae (order: Passeriformes) [59,61]. This complicated evolution of CRs of *Cacatua* species could be explained by a scenario in which most regions of duplicated CRs underwent a concerted evolution, while the 5′ and 3′ ends of the duplicated CRs evolved independently [59,61]. To analyze the pattern of the evolutionary scenario of CRs among the *Cacatua* species, the sequence similarity and phylogenetic relationship of paralogous CRs of *Cacatua* species were analyzed for each of the three domains in the CRs. Domain I, II, and III cover the 5′ end, middle, and 3′ end of CRs, respectively. In the sequence comparison, domain II showed the highest sequence similarity, while domain III showed the lowest sequence similarity between paralogous CRs (Supplementary Table S8). Domains I and II showed a similar size among the CRs of *Cacatua* species. In domain III, the size was very variable between CR1 and CR2 of five *Cacatua* species. In addition, most regions of domain III showed a high sequence similarity between paraglous CRs, while the 3′ end showed a very low sequence similarity between paralogous CRs. Additionally, in the phylogenetic analysis based on domain I, paralogous CRs of four *Cacatua* species were clustered together and paralogous CRs of C. *goffiniana* were not clustered (Supplementary Figure S3). Moreover, all paralogous CRs were clustered in phylogenetic trees for domain II (Supplementary Figure S4). Due to the difficulty in sequence alignment between the 3′ end of domain III, these sequences were eliminated in the phylogenetic analysis. In the phylogeny, paralogous CRs of five *Cacatua* species were clustered together (Supplementary Figures S5 and S6). Based on the sequence similarity and phylogenetic analysis, it is possible that domain I of the duplicated CRs in the *Cacatua* species evolved independently, while domain II experienced a concerted evolution. The evolutionary pattern of domain III was more complex than the other two domains. In domain III, most of the regions likely underwent a concerted evolution, while the 3′ end likely evolved independently. The evolutionary pattern of duplicated CRs of *Cacatua* species was consistent with an evolutionary scenario in families Aegithalidae and Zosteropidae, as suggested by the previous papers [59,61]. Nevertheless, it is worth noting that only five species of twelve *Cacatua* species recorded in the world were analyzed in this study. Therefore, additional mitogenomes from the remaining *Cacatua* species should be sequenced for testing the accurate evolutionary hypotheses of CRs in the *Cacatua* genus in future studies.

### 3.6. Gene Arrangements

**Figure 4:**
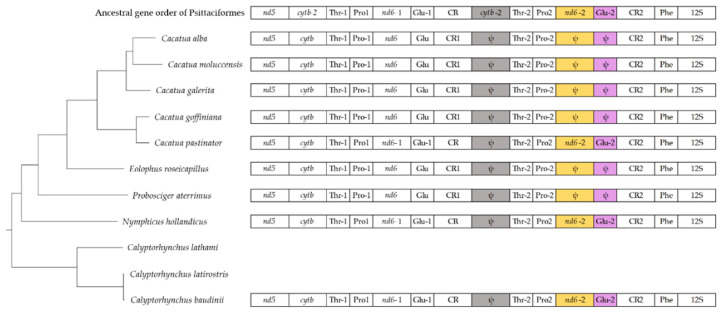
Gene arrangements of mitogenomes of Cacatuidae. The relationship of the species was based on a phylogenetic analysis. The gene order of mitogenome shows the region between *nd5* to 12S rRNA gene. Gene orders of *Calyptorhynchus lathami* and *Calyptorhynchus latirostris* are not shown since their complete mitogenomes have no duplicated region. Abbreviations: *nd5*: NADH dehydrogenase subunit 5; *cytb*: Cytochrome b; Thr: tRNA-Thr; Pro: tRNA-Pro; *nd6*: NADH dehydrogenase subunit 6; Glu: tRNA-Glu; CR: Control region; Phe: tRNA-Phe; 12S: 12S rRNA; ψ,: Pseudogene.

The gene order of the *Cacatua* mitogenome was the same across the species except for the region located between *nd5* and the 12S rRNA gene (Figure 4). Due to this difference, there were two types of gene arrangements in the *Cacatua* genus. These differences between the two types are color-coded in Figure 4. The first type had a degenerated *cytb*, *nd6,* and tRNA-Glu. The second type had a degenerated *cytb* and fully functional *nd6* and tRNA-Glu, which were more similar to the ancestral mitogenome of Psittaciformes [20]. The three species of the present study and *C. moluccensis* [20] were equivalent to the first type, while *C. pastinator* [20] was equivalent to the second type. In a previous study, the ancestral mitogenome of Psittaciformes revealed that the second copy of *cytb*, *nd6,* and tRNA-Glu are fully functional [21]. Moreover, the authors suggested that the first type mitogenome is derived from the second type mitogenome by degeneration [20]. Although complete mitogenomes of *Calyptorhynchus lathami* and *Calyptorhynchus latirostris* have been reported to have no duplicated regions [26], the recent study confirms that these mitogenomes include duplication [20]. Assuming that mitogenomes of *Calyptorhynchus lathami* and *Calyptorhynchus latirostris* were of the second type, this hypothesis [20] was reasonable.

### 3.7. Phylogenetic Analysis and Molecular Dating

**Figure 5:**
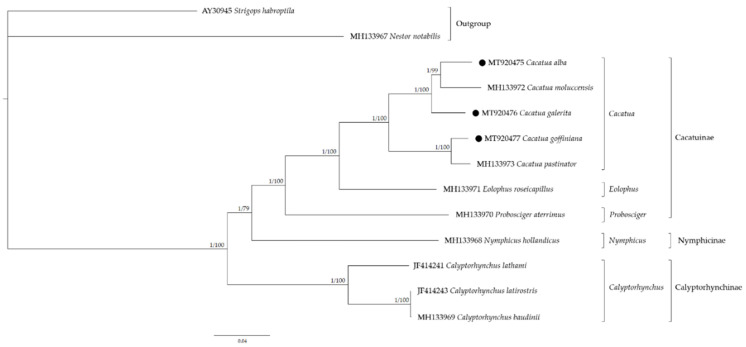
Phylogenetic tree of Cacatuidae according to 37 mitochondrial genes. The left value at the node is Bayesian inference (BI) posterior probability values and the right value at the node is Maximum likelihood (ML) bootstrap percentages. Black circles indicate the newly sequenced mitogenomes.

Phylogenetic relationships within the Cacatuidae were analyzed using 37 mitochondrial genes (Figure 5). Based on the phylogenetic tree, *Cacatua* formed a well-supported monophyletic lineage sister to *Eolophus roseicapilus*. The family Cacatuidae was divided into three subfamilies: Cacatuinae, Calyptorhynchinae, and Nymphicinae. The subfamily Cacatuinae, including *Cacatua* represented a crown group and Calyptorhynchinae represented the basal group within the Cacatuidae. *Cacatua* represented a crown group in the Cacatuidae and *C. galerita* was a basal group relative to the rest of the sampled *Cacatua* on this branch of the mitochondrial tree (*C. alba* and *C. moluccensis*). These phylogenetic relationships within Cacatuidae were similar to the previous study [20]. As a result of molecular dating (Supplementary Figure S7), the divergence of Cacatuidae and Psittacidae was estimated at 40.1 Mya, ranging from 28.9 to 50.5 Mya with a 95% HPD interval, similar to the previous studies [26,62,63,64]. Calyptorhynchinae diverged from another subfamily estimated at 27.0 Mya (range, 19.1~35.4 Mya; 95% HPD), followed by Nymphicinae, which diverged at 24.6 Mya (range, 16.5~32.1 Mya; 95% HPD), and Cacatuinae, which diverged at 20.5 Mya (range, 13.6~27.6 Mya; 95% HPD). The MRCA of *Cacatua* genus was estimated at about 10.2 Mya ranging from 6.1 to 14.7 Mya with a 95% HPD interval. The estimated evolution time of Cacatuidae was similar to that of fossil data involving *Cacatua* from Riversleigh deposits [65]. The adaptive radiation within the genus *Cacatua* occurred between the late Miocene and Pliocene. In Miocene, sclerophyll, eucalyptus, and grasslands were expanded in Australia and played an important role in the speciation of cockatoos [26]. Furthermore, it was suggested that cockatoos migrated between south-east Asia and Australia and diversified in the early to middle Pliocene [26].

## 4. Conclusions

In this study, the complete mitogenomes of *C. alba*, *C. galerita,* and *C. goffiniana* were newly described and compared among the *Cacatua* species. In addition, a detailed analysis of CRs of the *Cacatua* species was performed. Ten conserved motifs were found in both duplicated CRs of the *Cacatua* species. A sequence similarity and phylogenetic analysis for each of the three domains of duplicated CRs of the *Cacatua* species indicated that domain I and the 3′ end of domain III evolved independently, while domain II and most of the regions of domain III experienced a concerted evolution. The results of our phylogenetic analysis supported *Cacatua* as a single monophyletic taxonomic group. Molecular dating results were congruent with previous studies which showed that the *Cacatua* speciation occurs in Miocene and Pliocene. Data from our study might be useful for the detection of an illegal trade and the establishment of management units for conservation of *Cacatua* species. Especially, *C. alba* which is listed as an Endangered category in the IUCN Red List of Threatened Species needs a powerful conservation strategy to protect it from extinction. Compared to *C. alba*, *C. galerata* and *C. goffiniana* are less threatened, but need an accurate identification since they are commonly traded as pets in markets. Our detailed data of 13 protein-coding genes, two ribosomal RNA genes, and control regions in mitochondrial genomes, in the present study, might be helpful to provide specific markers for accurate identification and population studies of three focal species.

## Figures and Tables

**Table 1:** Species list and GenBank accession numbers of retrieved mitogenomes in this study.

Family	Subfamily	Species Name	Accession number	References
Cacatuidae	Cacatuinae	*C. alba*	MT920475	This study
		*C.galerita*	MT920476	This study
		*C. goffiniana*	MT920477	This study
		*C. moluccensis*	MH133972	[20]
		*C. pastinator*	MH133973	[20]
		*Eolophus roseicapillus*	MH133971	[20]
		*Probosciger aterrimus*	MH133970	[20]
	Calyptorhynchinae	*Calyptorhynchus baudinii*	MH133969	[20]
		*Calyptorhynchus lathami*	JF414241	[26]
		*Calyptorhynchus latirostris*	JF424243	[26]
	Nymphicinae	*Nymphicus hollandicus*	MH133968	[20]
Psittacidae		*Poicephalus gulielmi*	MF977813	[42]
		*Psittacus erithacus*	KM611474	[19]
Strigopidae		*Nestor notabilis*	MH133967	[20]
		*Strigops habroptila*	AY309456	[43]

**Table 2:** Nucleotide compositions of the mitochondrial genome of three species in this study.

Nucleotide Sequence		*C. alba*	*C. galerita*	*C. goffiniana*
Whole sequence	Length (bp)	18,894	18,900	19,084
	A (%)	29.5	29.4	29.7
	C (%)	31.6	31.8	31.3
	G (%)	15.0	15.1	14.8
	T (%)	24.0	23.7	24.2
	A + T (%)	53.5	53.1	53.9
Protein coding genes	Length (bp)	11,402	11,399	11,402
	A (%)	29.1	29.1	29.5
	C (%)	34.0	34.2	34.0
	G (%)	12.9	13.0	12.6
	T (%)	23.9	23.6	24.0
	A + T (%)	53.0	52.7	53.5
Ribosomal RNA genes	Length (bp)	2543	2542	2537
	A (%)	32.8	32.3	33.1
	C (%)	29.8	29.7	29.3
	G (%)	18.9	19.3	18.7
	T (%)	18.6	18.7	18.9
	A + T (%)	51.4	51.0	52.0
Transfer RNA genes	Length (bp)	1669	1673	1672
	A (%)	32.5	32.4	33.1
	C (%)	25.5	26.0	25.5
	G (%)	17.1	17.1	16.5
	T (%)	24.7	24.5	24.9
	A + T (%)	57.3	56.9	58.0
Control regions	Length (bp)	2428	2430	2591
	A (%)	22.6	22.6	21.9
	C (%)	25.8	26.1	24.7
	G (%)	18.9	19.1	19.3
	T (%)	32.8	32.2	34.0
	A + T (%)	55.4	54.8	55.9

**Table 3:** Lengths of three degenerated genes and similarities between functional and degenerated genes of five species of Cacatua genus.

Species	*cytb*			*nd6*			tRNA-Glu			References
	Functional gene (bp)	Degenerated gene (bp)	Sequence Similarity (%)	Functional gene (bp)	Degenerated gene (bp)	Sequence Similarity (%)	Functional gene (bp)	Degenerated gene (bp)	Sequence Similarity (%)	
*C. alba*	1140	114	92.1	519	650	92.9	75	50	50.0	This study
*C. galerita*	1140	115	88.7	519	653	92.4	75	50	41.8	This study
*C. goffiniana*	1140	113	94.6	519	680	90.8	75	51	41.5	This study
*C. moluccensis*	1140	115	92.2	507	644	95.0	75	35	60.6	[20]
C. pastinator *	1140	115	99.1	519	519	100	75	75	100	[20]

* *C. pastinator* had two copies of functional *nd6* and tRNA-Glu.

## Data Availability

The data presented in this study are available in the NCBI GenBank (accession number: MT920475-MT920477).

## Supplementary Materials

The following are available online at https://www.mdpi.com/2073-4425/12/2/209/s1. Figure S1: Phylogenetic tree of Cacatuidae according to control regions using the Bayesian inference (BI) method; Supplementary Figure S2: Phylogenetic tree of Cacatuidae according to control regions using the Maximum likelihood (ML) method; Supplementary Figure S3: Phylogenetic tree of *Cacatua* according to domain I of control regions; Supplementary Figure S4: Phylogenetic tree of *Cacatua* according to domain II of control regions; Figure S5: Phylogenetic tree of *Cacatua* according to domain III of control regions using the Bayesian inference (BI) method; Figure S6: Phylogenetic tree of *Cacatua* according to domain III of control regions using the Maximum likelihood (ML) method; Figure S7: Estimations of molecular data from the 37 genes of mitogenomes; Table S1: Primer sets for the sequencing duplicated region; Supplementary Table S2: Models used in Mr. Bayes program searched from the PartitionFinder 2 program; Supplementary Table S3: Mitochondrial genome organization of *C. alba*; Supplementary Table S4: Mitochondrial genome organization of *C. galerita*; Table S5: Mitochondrial genome organization of *C. goffiniana*; Table S6: The skew values of AT and GC in the mitogenomes of Cacatuidae; Table S7: Conserved motifs in three domains of control regions of *Cacatua* species; Table S8: Size and similarity of three domains of control regions of *Cacatua* species.
