# Supplementary material for: Characterization and Comparative Analysis of Complete Mitogenomes of Three Cacatua Parrots (Psittaciformes: Cacatuidae)
Source: Genes (Basel). 2021 Jan 31;12(2):209. doi: 10.3390/genes12020209 (PMC7910981; doi:10.3390/genes12020209)
Supplement: Supplementary file 1 [file genes-12-00209-s001.pdf]

**Table S1.** Primer sets for sequencing duplicated region.

| Primer name | Sequence (5′–3′) | Reference |
|---|---|---|
| CATd-F2 | ATGGTCGGCGGCCTGGTTCTGGTCCT | [20] |
| CATd-R3 | CATTAAGATGTCGCGATYACGAGGCA | [20] |
| KAKCR1-F1 | ATGGTCGGCGGCCTGGTTCTGGTCCT | [20] |
| KAKCR2-R3 | CATTAAGATGTCGCGATYACGAGGCA | [20] |
| Cas1F | TTGTTTGTTATTCACCAAACCCC | This study |
| Cas1R | TTGAATGCCCGATACACATAGC | This study |
| Cg1F | GCCTTCTTGTTGGCGTTGTTTA | This study |
| Cg1R | CGCACCCCCGTTAAAACAAT | This study |
| CD4F | TCTCGCTTGGTCTCTACCCA | This study |
| CD4R | TCCCGACTTCGTTTTGACGA | This study |
| CD5F | CTACACCCTCGCCCTACTTG | This study |
| CD5R | ACTGGAGTGCGGATACTTGC | This study |

**Table S2.** Models used in Mr. Bayes program searched from PartitionFinder 2 program.

| **Best-fit model** | **Nucleotide position** |
|---|---|
| GTR+I+G | 12S rRNA, 16S rRNA, *atp6*_1st, *atp8*_2nd, *cox1*_1st, *cox1*_3rd, *cox2*_1st, *cox2*_2nd, *cox2*_3rd, *cox3*_1st, *cox3*_2nd, *cox3*_3rd, *cytb*_1st, *cytb*_2nd, *cytb*_3rd, *nd1*_2st, *nd1*_2nd, *nd1*_3rd, *nd2*_3rd, *nd3*_1st, *nd3*_3rd *nd4*_1st, *nd4*_2nd, *nd4*_3rd, *nd4l*_2nd, *nd4l*_3rd, *nd5*_1st, *nd5*_2nd, *nd5*_3rd, tRNA |
| GTR+G | *atp8*_1st, *nd6*_1st, *nd6*_3rd |
| HYK+I+G | *atp6*_2nd, *atp6*_3rd, *nd2*_2nd, *nd3*_2nd, |
| HYK+G | *atp8*_2nd, *cox1*_2nd, *nd4l*_1st, *nd2*_1st, *nd6*_2nd |

**Table S3.** Mitochondrial genome organization of *C. alba.*

| Gene/region | Position | | Size | Codon | | Anticodon | Intergenic Region | Strand |
|---|---|---|---|---|---|---|---|---|
| | Start | Stop | | Initiation | Termination | | | |
| tRNA-Phe | 1 | 64 | 64 | | | GAA | | + |
| 12S rRNA | 65 | 1,033 | 969 | | | | | + |
| tRNA-Val | 1,034 | 1,105 | 72 | | | TAC | | + |
| 16S rRNA | 1,106 | 2,679 | 1,574 | | | | | + |
| tRNA-Leu | 2,680 | 2,754 | 75 | | | TAA | | + |
| *nd1* | 2,763 | 3,743 | 981 | ATG | AGG | | 8 | + |
| tRNA-Ile | 3,742 | 3,813 | 72 | | | GAT | -2 | + |
| tRNA-Gln | 3,821 | 3,891 | 71 | | | TTG | 7 | - |
| tRNA-Met | 3,891 | 3,959 | 69 | | | CAT | -1 | + |
| *nd2* | 3,960 | 4,999 | 1,040 | ATG | TA(A) | | | + |
| tRNA-Trp | 5,000 | 5,070 | 71 | | | TCA | | + |
| tRNA-Ala | 5,072 | 5,140 | 69 | | | TGC | 1 | - |
| tRNA-Asn | 5,142 | 5,215 | 74 | | | GTT | 1 | - |
| tRNA-Cys | 5,218 | 5,284 | 67 | | | GCA | 2 | - |
| tRNA-Tyr | 5,285 | 5,354 | 70 | | | GTA | | - |
| *cox1* | 5,364 | 6,911 | 1,548 | GTG | AGG | | 9 | + |
| tRNA-Ser | 6,912 | 6,978 | 67 | | | TGA | | - |
| tRNA-Asp | 6,983 | 7,051 | 69 | | | GTC | 4 | + |
| *cox2* | 7,054 | 7,737 | 684 | ATG | TAA | | 2 | + |
| tRNA-Lys | 7,739 | 7,806 | 68 | | | TTT | 1 | + |
| *atp8* | 7,808 | 7,975 | 168 | ATG | TAG | | 1 | + |
| *atp6* | 7,966 | 8,648 | 683 | ATG | TA(A) | | -10 | + |
| *cox3* | 8,649 | 9,432 | 784 | ATG | T(AA) | | | + |
| tRNA-Gly | 9,433 | 9,501 | 69 | | | TCC | | + |
| *nd3-α* | 9,502 | 9,674 | 173 | ATA | | | | + |
| *nd3-β* | 9,676 | 9,852 | 177 | | TA(A) | | 1 | + |
| tRNA-Arg | 9,853 | 9,920 | 68 | | | TCG | | + |
| *nd4l* | 9,922 | 10,218 | 297 | ATG | TAA | | 1 | + |
| *nd4* | 10,212 | 11,604 | 1,393 | ATG | T(AA) | | -7 | + |
| tRNA-His | 11,605 | 11,673 | 69 | | | GTG | | + |
| tRNA-Ser | 11,674 | 11,739 | 66 | | | GCT | | + |
| tRNA-Leu | 11,740 | 11,809 | 70 | | | TAG | | + |
| *nd5* | 11,810 | 13,624 | 1,815 | ATG | TAA | | | + |
| *cytb* | 13,637 | 14,776 | 1,140 | ATG | TAA | | 12 | + |
| tRNA-Thr | 14,777 | 14,844 | 68 | | | TGT | | + |
| tRNA-Pro | 14,846 | 14,914 | 69 | | | TGG | 1 | - |
| *nd6* | 14,918 | 15,436 | 519 | ATG | TAG | | 3 | - |
| tRNA-Glu | 15,437 | 15,511 | 75 | | | TTC | | - |
| Control region1 | 15,512 | 16,697 | 1,186 | | | | | + |
| degenerated *cytb* | 16,698 | 16,811 | 114 | | | | | + |
| tRNA-Thr | 16,812 | 16,879 | 68 | | | TGT | | + |
| tRNA-Pro | 16,881 | 16,949 | 69 | | | TGG | 1 | - |
| degenerated *nd6* | 16,953 | 17,602 | 650 | | | | 3 | - |
| degenerated tRNA-Glu | 17,603 | 17,652 | 50 | | | | | - |
| Control region2 | 17,653 | 18,894 | 1,242 | | | | | + |

**Table S4.** Mitochondrial genome organization of *C. galerita.*

| Gene/region | Position | | Size | Codon | | Anticodon | Intergenic Region | Strand |
|---|---|---|---|---|---|---|---|---|
| | Start | Stop | | Initiation | Termination | | | |
| tRNA-Phe | 1 | 67 | 67 | | | GAA | | + |
| 12S rRNA | 68 | 1,036 | 969 | | | | | + |
| tRNA-Val | 1,037 | 1,108 | 72 | | | TAC | | + |
| 16S rRNA | 1,109 | 2,681 | 1,573 | | | | | + |
| tRNA-Leu | 2,682 | 2,757 | 76 | | | TAA | | + |
| *nd1* | 2,766 | 3,746 | 981 | ATG | AGG | | 8 | + |
| tRNA-Ile | 3,745 | 3,816 | 72 | | | GAT | -2 | + |
| tRNA-Gln | 3,824 | 3,894 | 71 | | | TTG | 7 | - |
| tRNA-Met | 3,894 | 3,962 | 69 | | | CAT | -1 | + |
| *nd2* | 3,963 | 5,002 | 1,040 | ATG | TA(A) | | | + |
| tRNA-Trp | 5,003 | 5,073 | 71 | | | TCA | | + |
| tRNA-Ala | 5,075 | 5,143 | 69 | | | TGC | 1 | - |
| tRNA-Asn | 5,145 | 5,218 | 74 | | | GTT | 1 | - |
| tRNA-Cys | 5,221 | 5,287 | 67 | | | GCA | 2 | - |
| tRNA-Tyr | 5,288 | 5,357 | 70 | | | GTA | | - |
| *cox1* | 5,367 | 6,914 | 1,548 | GTG | AGG | | 9 | + |
| tRNA-Ser | 6,915 | 6,981 | 67 | | | TGA | | - |
| tRNA-Asp | 6,986 | 7,054 | 69 | | | GTC | 4 | + |
| *cox2* | 7,057 | 7,740 | 684 | ATG | TAA | | 2 | + |
| tRNA-Lys | 7,742 | 7,809 | 68 | | | TTT | 1 | + |
| *atp8* | 7,811 | 7,975 | 165 | ATG | TAA | | 1 | + |
| *atp6* | 7,966 | 8,648 | 683 | ATG | TA(A) | | -10 | + |
| *cox3* | 8,649 | 9,432 | 784 | ATG | T(AA) | | | + |
| tRNA-Gly | 9,433 | 9,501 | 69 | | | TCC | | + |
| *nd3-α* | 9,502 | 9,674 | 173 | ATA | | | | + |
| *nd3-β* | 9,676 | 9,852 | 177 | | TA(A) | | 1 | + |
| tRNA-Arg | 9,853 | 9,920 | 68 | | | TCG | | + |
| *nd4l* | 9,922 | 10,218 | 297 | ATG | TAA | | 1 | + |
| *nd4* | 10,212 | 11,604 | 1,393 | ATG | T(AA) | | -7 | + |
| tRNA-His | 11,605 | 11,673 | 69 | | | GTG | | + |
| tRNA-Ser | 11,674 | 11,739 | 66 | | | GCT | | + |
| tRNA-Leu | 11,740 | 11,809 | 70 | | | TAG | | + |
| *nd5* | 11,810 | 13,624 | 1,815 | ATG | TAA | | | + |
| *cytb* | 13,637 | 14,776 | 1,140 | ATG | TAA | | 12 | + |
| tRNA-Thr | 14,777 | 14,844 | 68 | | | TGT | | + |
| tRNA-Pro | 14,846 | 14,914 | 69 | | | TGG | 1 | - |
| *nd6* | 14,918 | 15,436 | 519 | ATG | TAG | | 3 | - |
| tRNA-Glu | 15,437 | 15,511 | 75 | | | TTC | | - |
| Control region1 | 15,512 | 16,695 | 1,184 | | | | | + |
| degenerated *cytb* | 16,696 | 16,810 | 115 | | | | | + |
| tRNA-Thr | 16,811 | 16,878 | 68 | | | TGT | | + |
| tRNA-Pro | 16,880 | 16,948 | 69 | | | TGG | 1 | - |
| degenerated *nd6* | 16,952 | 17,604 | 653 | | | | 3 | - |
| degenerated tRNA-Glu | 17,605 | 17,654 | 50 | | | | | - |
| Control region2 | 17,655 | 18,900 | 1,246 | | | | | + |

**Table S5.** Mitochondrial genome organization of *C. goffiniana.*

| Gene/region | Position | | Size | Codon | | Anticodon | Intergenic Sequence | Strand |
|---|---|---|---|---|---|---|---|---|
| | Start | Stop | | Initiation | Termination | | | |
| tRNA-Phe | 1 | 66 | 66 | | | GAA | | + |
| 12S rRNA | 67 | 1,032 | 966 | | | | | + |
| tRNA-Val | 1,033 | 1,104 | 72 | | | TAC | | + |
| 16S rRNA | 1,105 | 2,675 | 1,571 | | | | | + |
| tRNA-Leu | 2,676 | 2,751 | 76 | | | TAA | | + |
| *nd1* | 2,760 | 3,740 | 981 | ATG | AGG | | 8 | + |
| tRNA-Ile | 3,739 | 3,810 | 72 | | | GAT | -2 | + |
| tRNA-Gln | 3,818 | 3,888 | 71 | | | TTG | 7 | - |
| tRNA-Met | 3,888 | 3,956 | 69 | | | CAT | -1 | + |
| *nd2* | 3,957 | 4,996 | 1,040 | ATG | TA(A) | | | + |
| tRNA-Trp | 4,997 | 5,067 | 71 | | | TCA | | + |
| tRNA-Ala | 5,069 | 5,137 | 69 | | | TGC | 1 | - |
| tRNA-Asn | 5,139 | 5,212 | 74 | | | GTT | 1 | - |
| tRNA-Cys | 5,215 | 5,281 | 67 | | | GCA | 2 | - |
| tRNA-Tyr | 5,282 | 5,351 | 70 | | | GTA | | - |
| *cox1* | 5,361 | 6,908 | 1,548 | GTG | AGG | | 9 | + |
| tRNA-Ser | 6,909 | 6,975 | 67 | | | TGA | | - |
| tRNA-Asp | 6,980 | 7,048 | 69 | | | GTC | 4 | + |
| *cox2* | 7,051 | 7,734 | 684 | ATG | TAA | | 2 | + |
| tRNA-Lys | 7,736 | 7,803 | 68 | | | TTT | 1 | + |
| *atp8* | 7,805 | 7,972 | 168 | ATG | TAA | | 1 | + |
| *atp6* | 7,963 | 8,645 | 683 | ATG | TA(A) | | -10 | + |
| *cox3* | 8,646 | 9,429 | 784 | ATG | T(AA) | | | + |
| tRNA-Gly | 9,430 | 9,498 | 69 | | | TCC | | + |
| *nd3-α* | 9,499 | 9,671 | 173 | ATA | | | | + |
| *nd3-β* | 9,673 | 9,849 | 177 | | TA(A) | | 1 | + |
| tRNA-Arg | 9,850 | 9,917 | 68 | | | TCG | | + |
| *nd4l* | 9,919 | 10,215 | 297 | ATG | TAA | | 1 | + |
| *nd4* | 10,209 | 11,601 | 1,393 | ATG | T(AA) | | -7 | + |
| tRNA-His | 11,602 | 11,670 | 69 | | | GTG | | + |
| tRNA-Ser | 11,671 | 11,736 | 66 | | | GCT | | + |
| tRNA-Leu | 11,737 | 11,806 | 70 | | | TAG | | + |
| *nd5* | 11,807 | 13,621 | 1,815 | ATG | TAA | | | + |
| *cytb* | 13,634 | 14,773 | 1,140 | ATG | TAA | | 12 | + |
| tRNA-Thr | 14,774 | 14,841 | 68 | | | TGT | | + |
| tRNA-Pro | 14,843 | 14,911 | 69 | | | TGG | 1 | - |
| *nd6* | 14,915 | 15,433 | 519 | ATG | TAG | | 3 | - |
| tRNA-Glu | 15,434 | 15,508 | 75 | | | TTC | | - |
| Control region1 | 15,509 | 16,827 | 1,319 | | | | | + |
| degenerated *cytb* | 16,828 | 16,940 | 113 | | | | | + |
| tRNA-Thr | 16,941 | 17,008 | 68 | | | TGT | | + |
| tRNA-Pro | 17,010 | 17,078 | 69 | | | TGG | 1 | - |
| degenerated *nd6* | 17,082 | 17,761 | 680 | | | | 3 | - |
| degenerated tRNA-Glu | 17,762 | 17,812 | 51 | | | | | - |
| Control region2 | 17,813 | 19,084 | 1,272 | | | | | + |

**Table S6.** The skew values of AT and GC in the mitogenomes of Cacatuidae.

| Subfamily | Species | Accession number | Whole sequence | | PCGs | |
|---|---|---|---|---|---|---|
| | | | AT skew | GC skew | AT skew | GC skew |
| Cacatuinae | *Cacatua alba* | MT920475 | 0.102 | -0.357 | 0.099 | -0.449 |
| | *Cacatua galerita* | MT920476 | 0.108 | -0.356 | 0.104 | -0.448 |
| | *Cacatua goffiniana* | MT920477 | 0.102 | -0.358 | 0.103 | -0.457 |
| | *Cacatua moluccensis* | MH133972 | 0.106 | -0.350 | 0.101 | -0.444 |
| | *Cacatua pastinator* | MH133973 | 0.092 | -0.361 | 0.122 | -0.457 |
| | *Eolophus roseicapillus* | MH133971 | 0.118 | -0.374 | 0.113 | -0.482 |
| | *Probosciger aterrimus* | MH133970 | 0.120 | -0.392 | 0.121 | -0.488 |
| Calyptorhynchinae | *Calyptorhynchus baudinii* | MH133969 | 0.119 | -0.376 | 0.134 | -0.465 |
| | *Calyptorhynchus lathami* | JF414241 | 0.123 | -0.388 | 0.115 | -0.468 |
| | *Calyptorhynchus latirostris* | JF414243 | 0.128 | -0.387 | 0.117 | -0.465 |
| Nymphicinae | *Nymphicus hollandicus* | MH133968 | 0.134 | -0.409 | 0.161 | -0.508 |
| | Average | | 0.114 | -0.374 | 0.117 | -0.466 |

**Table S7.** Conserved motifs in three domains of control regions of *Cacatua* species.

| Domain | Conserved region | CRs | Initiation | Termination | Consensus sequence | Similarity (%) |
|---|---|---|---|---|---|---|
| Domain I | poly-C | CR1 | 15,532 | 15,549 | CCCCCCCCTTCCCCCCCC | 100 |
| | | CR2 | 17,855 | 17,872 | | |
| Domain II | ETAS1 | CR1 | 15,573 | 15,636 | ARGYTATGTGTATCGRGCATT-CARYARYKRTCCTYAWYACAYTTCATTCAST-BWHNDGTGGRYA | 85.3 |
| | | CR2 | 17,897 | 17,960 | | |
| | ETAS2 | CR1 | 15,645 | 15,691 | YATGBTCTATCYCATTDYNCYGYDYBBYT-GRATYGTTGGYGGTACMG | 83.3 |
| | | CR2 | 17,969 | 18,015 | | |
| | F-box | CR1 | 15,892 | 15,917 | ARCTCMCGAGAAATCAGCAACTGGAT | 96.6 |
| | | CR2 | 18,217 | 18,242 | | |
| | D-box | CR1 | 15,995 | 16,019 | TCTGGTTCCTCGGTCAGGGCCATRA | 98.6 |
| | | CR2 | 18,320 | 18,344 | | |
| | C-box | CR1 | 16,045 | 16,078 | CTCCACCGGGTCATTTGGTTCGCCCTTGGA-TAGC | 100 |
| | | CR2 | 18,370 | 18,403 | | |
| | BS-box | CR1 | 16,278 | 16,292 | CACTTTGYTTCGCAT | 96.4 |
| | | CR2 | 18,604 | 18,618 | | |
| | B-box | CR1 | 16,296 | 16,307 | GTTATGGYTMTC | 94.1 |
| | | CR2 | 18,622 | 18,633 | | |
| Domain III | CSB1 | CR1 | 16,339 | 16,365 | MATGRTTRYYAGACATAATTCTCTTAC | 91.4 |
| | | CR2 | 18,665 | 18,691 | | |
| | BTP | CR1 | 16,405 | 16,430 | GTCAAAACGAAGTCGGGAAATYTCTA | 98.6 |
| | | CR2 | 18,731 | 18,756 | | |

**Table S8.** Size and similarity of three domains of control regions of *Cacatua* species.

| **Species** | **Accession number** | **Domain I** | | **Domain II** | | **Domain III** | |
|---|---|---|---|---|---|---|---|
| | | **Size of CR1/CR2 (bp)** | **Similarity (%)** | **Size of CR1/CR2 (bp)** | **Similarity (%)** | **Size of CR1/CR2 (bp)** | **Similarity (%)** |
| *Cacatua alba* | MT920475 | 374/372 | 91.4 | 451/452 | 98.5 | 360/418 | 79.7 |
| *Cacatua galerita* | MT920476 | 372/371 | 94.1 | 451/453 | 98.2 | 361/422 | 89.6 |
| *Cacatua goffiniana* | MT920477 | 372/372 | 94.4 | 453/453 | 99.8 | 492/447 | 854 |
| *Cacatua moluccensis* | MH133972 | 375/374 | 89.1 | 451/452 | 98.9 | 360/417 | 79.4 |
| *Cacatua pastinator* | MH133973 | 372/372 | 98.1 | 453/453 | 100.0 | 497/535 | 85.1 |

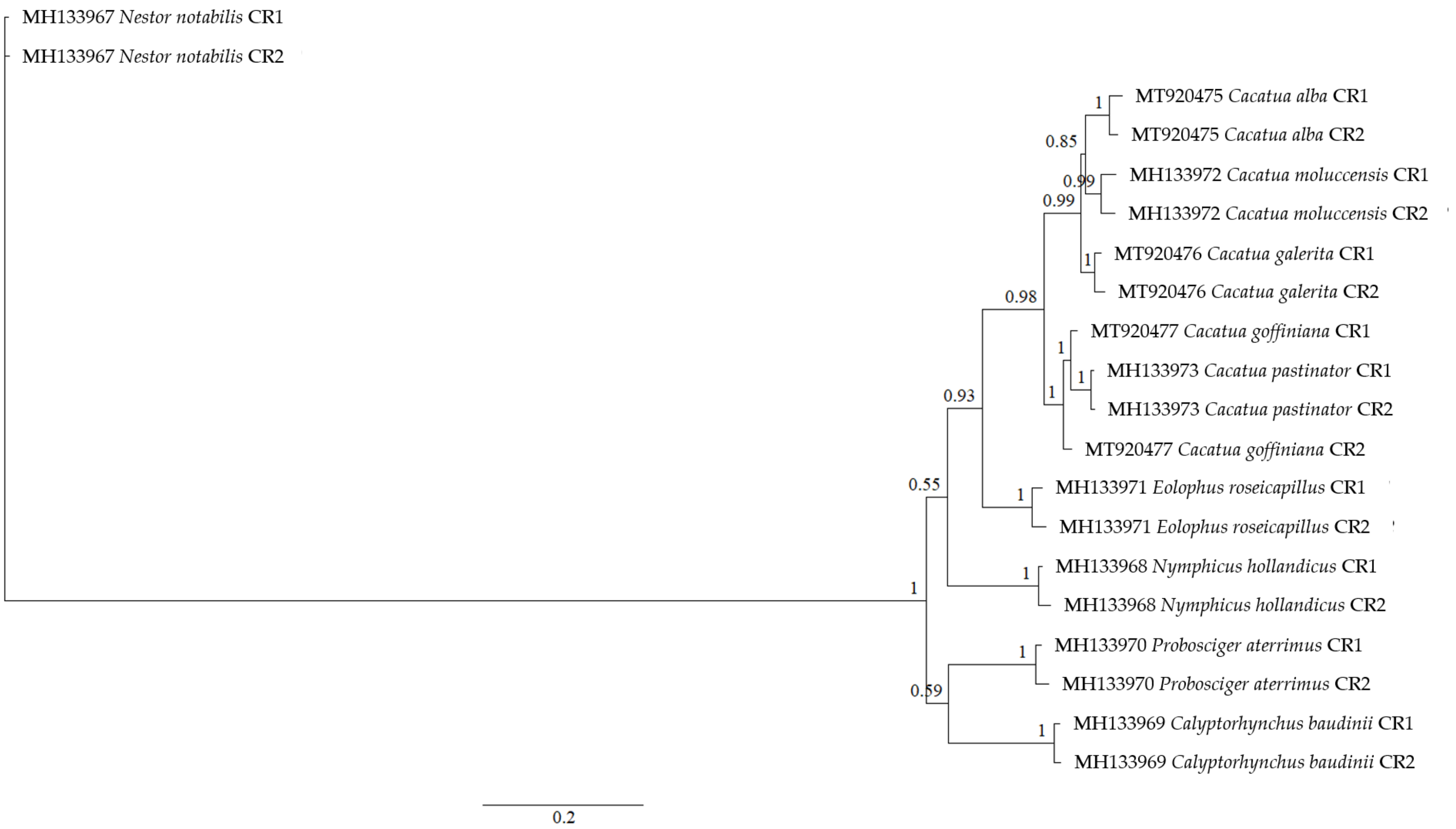


**Figure S1.** Phylogenetic tree of Cacatuidae according to control regions using the Bayesian inference (BI) method. The number at the internodes are BI posterior probability values.

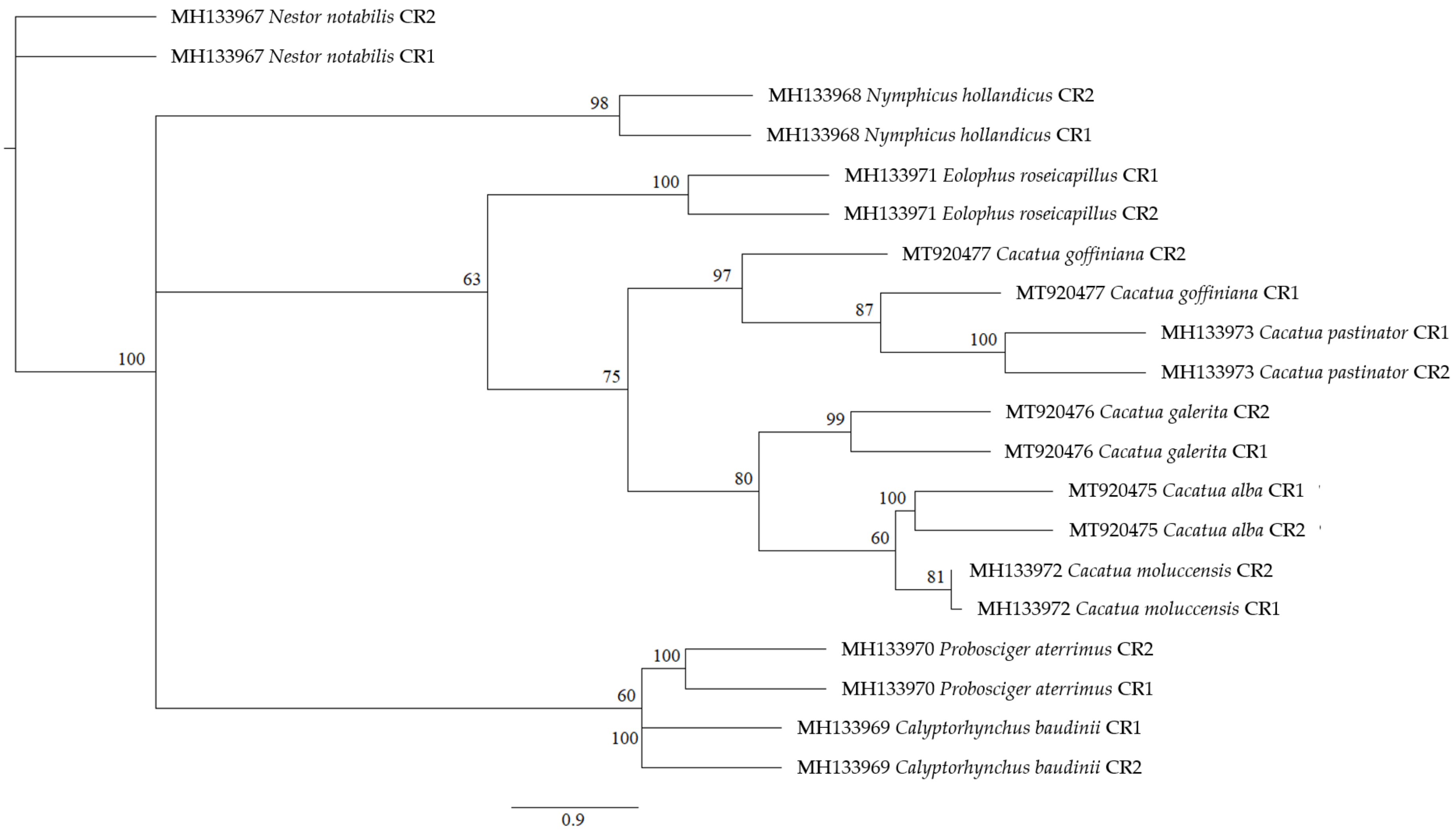


**Figure S2.** Phylogenetic tree of Cacatuidae according to control regions using the Maximum likelihood (ML) method. The number at the internodes are ML bootstrap percentages.

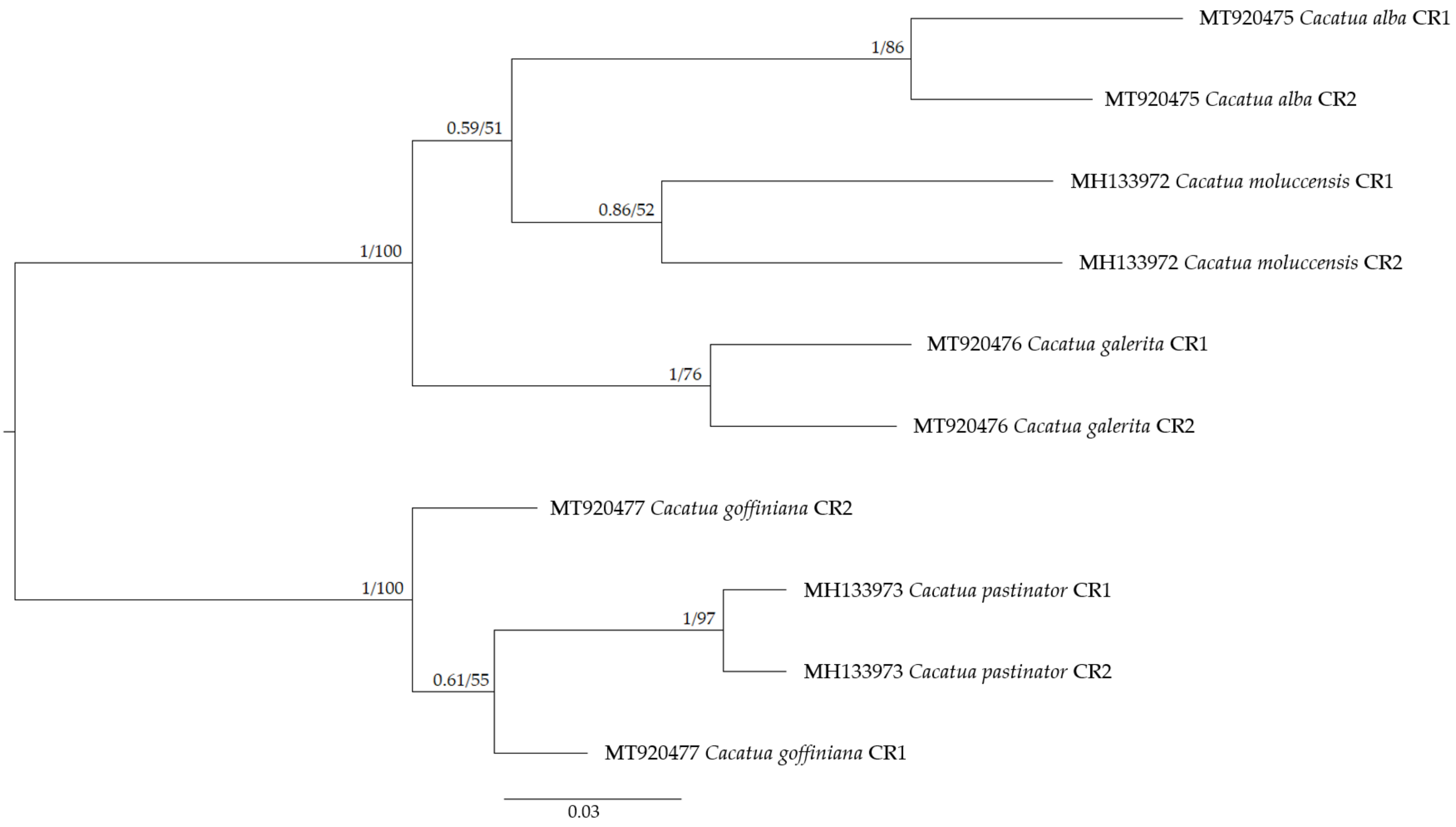


**Figure S3.** Phylogenetic tree of *Cacatua* according to domain I of control regions. The left value at the node is Bayesian Inference (BI) posterior probability values and the right value at the node is Maximum Likelihood (ML) bootstrap percentages.

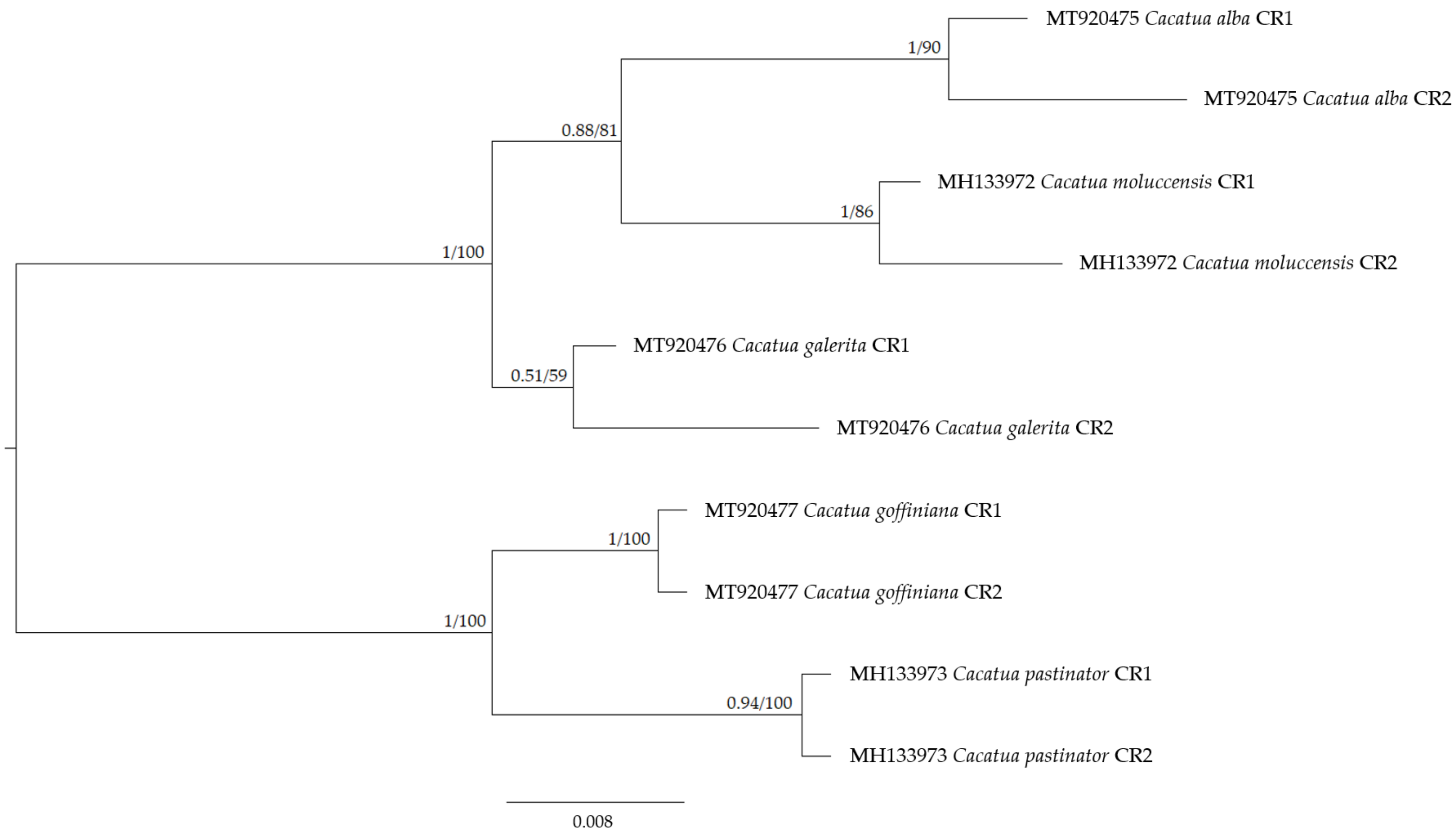


**Figure S4.** Phylogenetic tree of *Cacatua* according to domain II of control regions. The left value at the node is Bayesian Inference (BI) posterior probability values and the right value at the node is Maximum Likelihood (ML) bootstrap percentages.

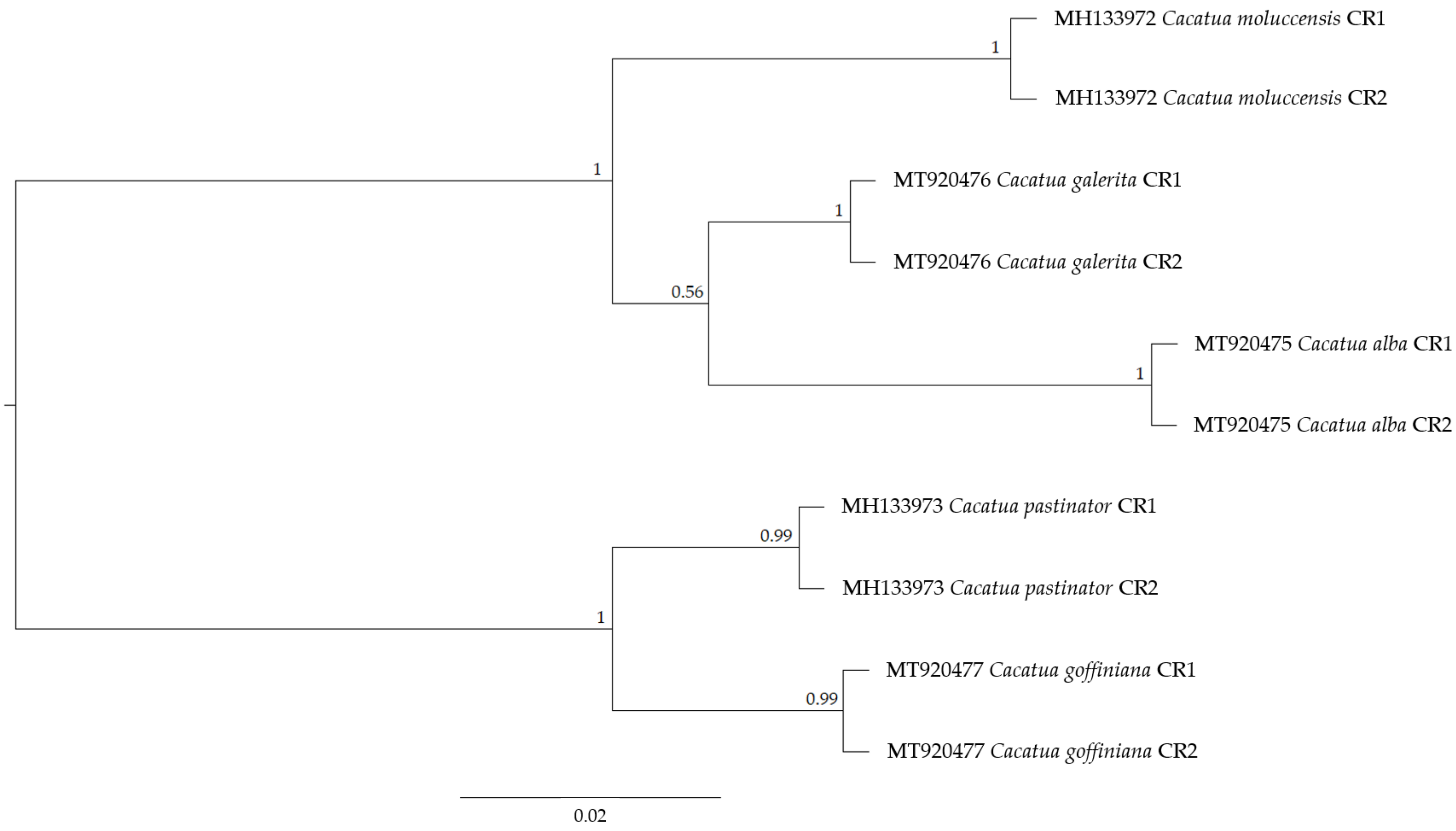


**Figure S5.** Phylogenetic tree of *Cacatua* according to domain III of control regions using the Bayesian inference (BI) method. The number at the internodes are BI posterior probability values.

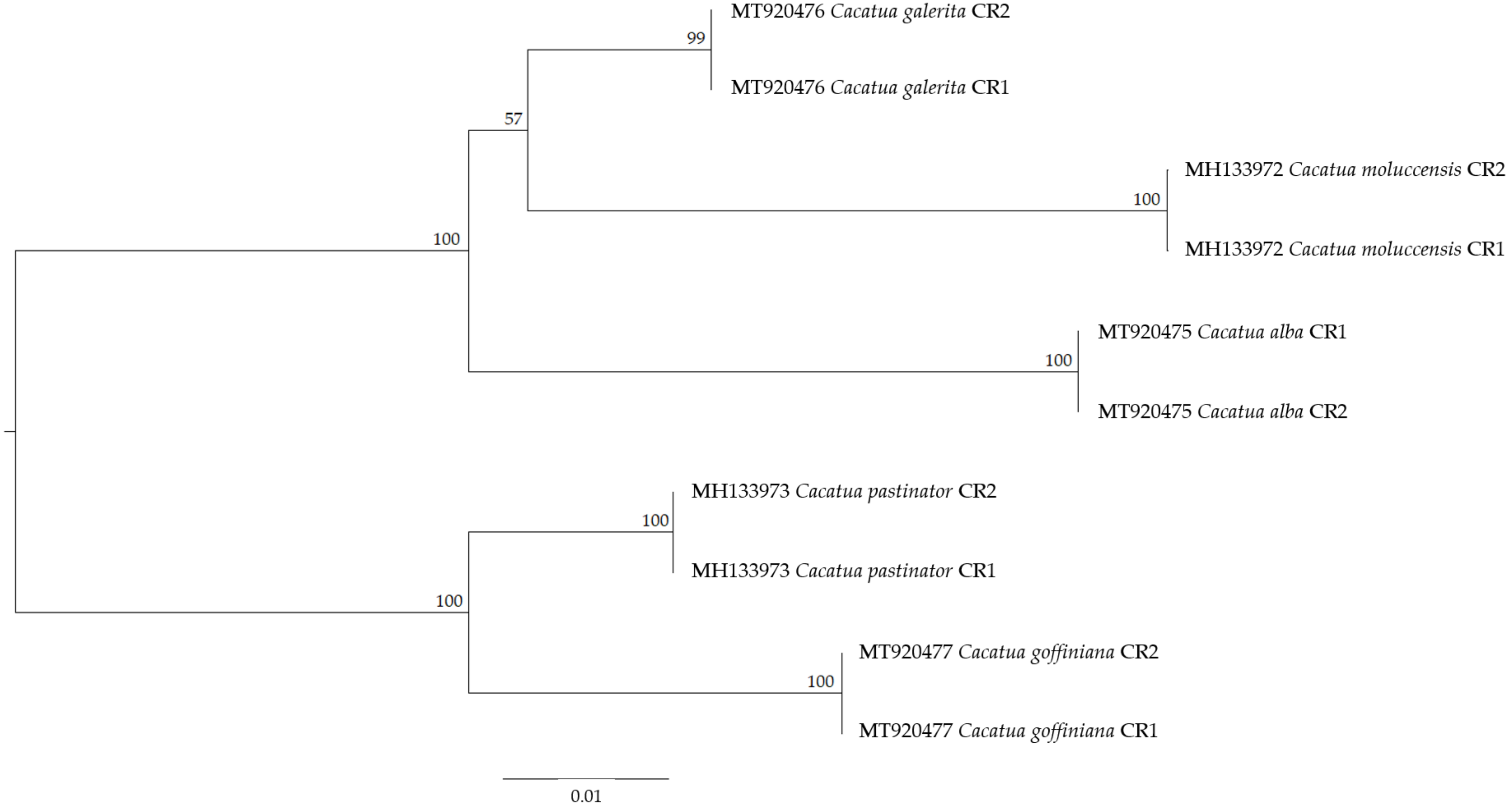


**Figure S6.** Phylogenetic tree of *Cacatua* according to domain III of control regions using the Maximum likelihood (ML) method. The number at the internodes are ML bootstrap percentages.

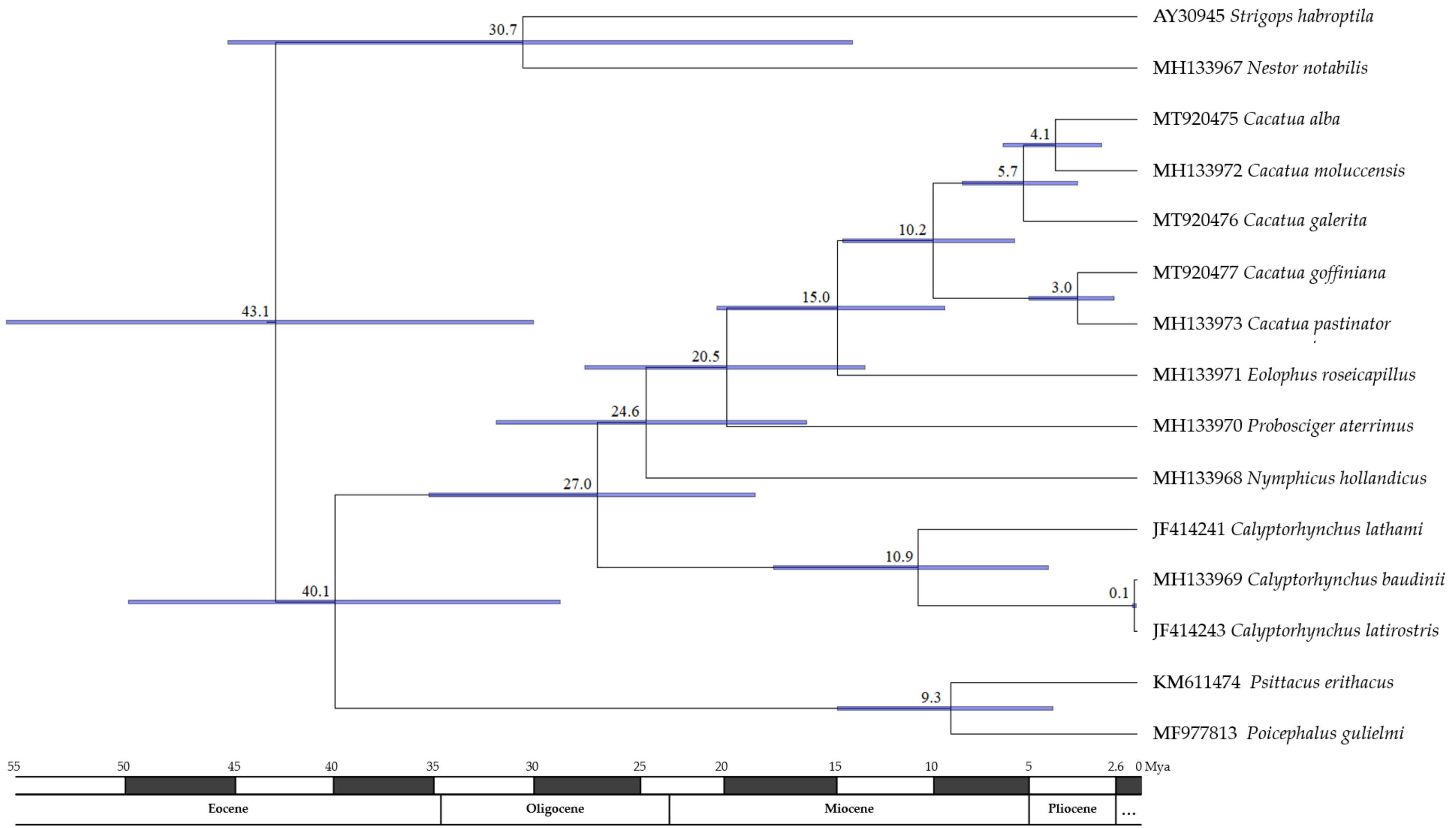


**Figure S7.** Estimations of molecular date from the 37 genes of mitogenomes. The values of node represent median age (million year ago, Mya). The blue bars represent estimated range of age (Mya) (95% highest posterior density; HPD
